# Protein kinase inhibitor responses in uveal melanoma reflects a diminished dependency on PKC-MAPK signaling

**DOI:** 10.1038/s41417-022-00457-2

**Published:** 2022-03-29

**Authors:** John J. Park, Ashleigh Stewart, Mal Irvine, Bernadette Pedersen, Zizhen Ming, Matteo S. Carlino, Russell J. Diefenbach, Helen Rizos

**Affiliations:** 1 Macquarie Medical School, Faculty of Medicine, Health and Human Sciences, Macquarie University, Sydney, Australia; 2 Melanoma Institute Australia, Sydney, Australia; 3 Department of Medical Oncology, Westmead and Blacktown Hospitals, Sydney, Australia

**Keywords:** Oncogenes, Cell biology

## Abstract

Uveal melanoma (UM) is a rare cancer arising from melanocytes in the uveal tract of the eye. Despite effective primary treatment, there is no approved therapy for metastatic UM and prognosis and survival remain poor. Over 90% of UM are driven by mutations affecting the Gα subunits encoded by the *GNAQ* and *GNA11* genes. These mutations activate downstream and targetable signaling pathways, including the protein kinase C (PKC) cascade. PKC inhibitors have been used in clinical trials for metastatic UM but have shown limited efficacy. In this study, we examined the signaling and functional effects of two PKC inhibitors (AEB071 and IDE196) in a panel of UM cell models. In response to PKC inhibition, all UM cell lines showed potent suppression of PKC activity, but this was not sufficient to predict PKC inhibitor sensitivity and only two UM cell lines showed substantial PKC inhibitor-induced cell death. The differences in UM cell responses to PKC inhibition were not attributable to the degree or timing of PKC suppression or inhibition of the downstream mitogen-activated protein kinase (MAPK) or phosphatidylinositol-3-kinase (PI3K) pathways. Instead, UM cell show complex, PKC-independent signaling pathways that contribute to their survival and resistance to targeted therapies.

## Introduction

Uveal melanoma (UM) is the most common primary ocular malignancy in adults, affecting 5–7 individuals per million per year [1, 2]. UM arises from melanocytes within the uveal tract, with more than 90% originating from the choroid followed by the iris and ciliary body [3]. Although primary UM can be treated successfully with surgery or radiation therapy, ~50% of patients will develop metastatic disease [4] with over 90% of metastases occurring in the liver [5]. Until recently, there was no effective systemic therapy for patients with metastatic UM, and outcomes remained poor. In a recent phase 3 trial, the bispecific protein tebentafusp (recognises the CD3-T-cell receptor and an HLA-A02:01 presented melanocytic antigen gp100) resulted in improved progression-free survival (PFS) and overall survival (OS) in previously untreated HLA-A02:01-positive advanced UM patients [6].

Over 95% of UM harbor mutually exclusive activating driver mutations in *GNAQ* or *GNA11* with another 6–8% of UM carrying mutations in the *CYSLTR2* or *PLCB4* genes [7–12]. These four gene products interact within a common pathway that is initiated upon ligand-activation of G protein-coupled receptors (GPCR), such as CYSLTR2. GPCRs bind to Gα proteins, which include the G_q_ and G_11_ subunits encoded by *GNAQ* and *GNA11*, respectively. These GTPase subunits transmit signals between GPCRs and downstream molecules such as the canonical effector phospholipase C-β (PLC-β) (*PLCB4*). PLC-ß promotes the sequential activation of secondary signaling molecules that stimulate protein kinase C (PKC) activity [13]. The stimulation of PKC activates multiple pathways including the mitogen-activated protein kinase (MAPK) cascade via the activation of the RAS exchange effector, RasGRP3 [14]. Importantly, GNAQ-mutant UM remain dependent on the initiating GNAQ mutations and appear to require PKC/MAPK signaling for survival [10, 15].

The activation of PKC by UM driver mutations highlighted PKC as a potential therapeutic target in UM. PKC is a family of isoforms that are subdivided into the classical PKCs (PKCα, PKCβ1, PKCβII, and PKCγ), novel PKCs (PKCδ, PKCε, PKCη, and PKCθ) and atypical PKCs (PKCζ and PKCι) [16]. These isoforms play critical roles in the regulation of cell proliferation, apoptosis, differentiation, angiogenesis and tumor development [16]. Two selective PKC inhibitors AEB071 and IDE196 (darovasertib, previously known as LXS196), have been trialed in the treatment of metastatic UM. AEB071, a potent inhibitor of the classical PKC isoforms (a, ß) and the novel PKC isoforms (δ, ε, η, θ), was evaluated in a phase 1 trial (NCT01430416) of 118 metastatic UM patients (96% with GNAQ/GNA11 mutations). AEB071 resulted in one patient with partial response (PR) and 55 patients (55/118; 47%) with stable disease (SD). The progression-free survival (PFS) was 15.4 weeks [17]. The second-generation PKC inhibitor, IDE196 inhibits the classical PKCα and novel PKCθ isoforms, and preliminary trial data (NCT02601378) confirmed only six partial responses (PRs) from 66 metastatic UM patients treated with single-agent IDE196 in dose escalation [18].

In order to reconcile the central role of PKC in GNAQ/GNA11 signaling with the poor response rates observed in PKC inhibitor monotherapy UM trials, we evaluated the effects of PKC inhibitors IDE196 and AEB071 in a large panel of UM cell lines. Both PKC inhibitors effectively suppressed PKC activity in all UM cell lines. Nevertheless, PKC inhibition resulted in variable downstream signaling changes and the MAPK pathway was only inhibited in the GNAQ/GNA11-mutant UM cell models. Critically, PKC inhibition was not sufficient to predict the degree of MAPK suppression or the response of UM to PKC inhibition. In fact, the differences in UM cell responses to PKC inhibition were not attributable to the degree or timing of PKC or MAPK inhibition, or the transcriptional inhibition of additional signaling cascades. This work confirms that PKC is not a central effector of UM survival and highlights the need for combination therapies targeting multiple signaling pathways in UM.

## Results

### PKC inhibitor activity does not predict downstream signaling responses in UM

We examined the effects of PKC inhibitors (IDE196 and AEB071) on a panel of 11 UM cell lines. These models included nine cell lines with a GNAQ or GNA11 mutation and two wild-type UM cell lines with no alterations in the *GNAQ* and *GNA11* genes (Table 1).

**Table 1:** Details of uveal melanoma cell lines.

Cell line	Origin	GNAQ	GNA11	EIF1AX	SF3B1	BAP1
92.1	Primary, choroid	Q209L (c.626A>T)	–	G6D c.17G>A	–	–
Mel202	Primary, choroid	Q209L (c.626A>T) & R210K (c.629G> A)	–	–	R625G c.1873C>G	–
MP46	Primary, PDX	Q209L (c.626A>T)	–	–	–	–
Mel270	Primary, ciliochoroidal	Q209P (c.626A>C)	–	–	–	–
MP38	Primary	Q209L (c.626A>T)	–	–	–	c.68–9_72del
OMM1.3	Metastatic, liver	Q209P (c.626A>C)	–	–	–	–
OMM1.5	Metastatic, liver	Q209P (c.626A>C)	–	–	–	–
MP41	Primary, PDX	–	Q209L (c.626A>T)	–	–	–
OMM1	Metastatic, subcutaneous	–	Q209L (c.626A>T)	–	–	–
Mel285	Primary, ciliochoroidal	–	–	–	–	–
Mel290	Primary, choroid	–	–	–	–	–

UM cell details derived from refs. [48–50]. No mutations in *CYSTLR2* and *PLCB4* have been reported in these UM cell lines.

*–* no mutation, *PDX* patient-derived xenograft models.

**Fig. 1:**
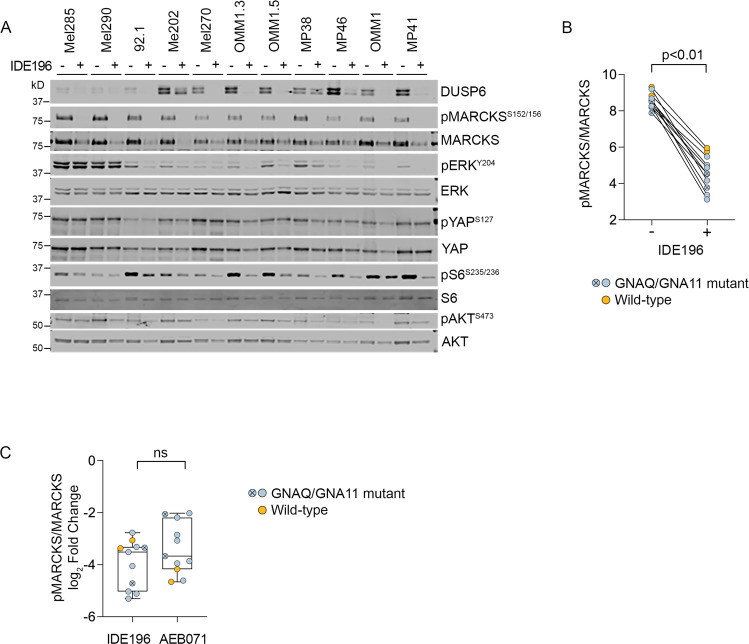
IDE196 suppresses MARCKS phosphorylation in UM cell lines. **A** Protein expression of MAPK, PI3K, YAP and PKC signaling effectors after 24 h of treatment with BSA control (−) or 5 µM IDE196 (+). **B** Normalized levels of pMARCKS were compared pre (−) and post (+) PKC inhibitor treatment using the paired t-test. Normalized MARCKS protein expression is derived from an average of 2–3 independent experiments for each cell line. The GNAQ/GNA11-mutant Mel270 and OMM1 cells are highlighted by the crossed, circle symbol. **C** The degree of pMARCKS inhibition (log_2_ fold change) in response to 24 h treatment with the PKC inhibitor IDE196 and AEB071 was compared using unpaired *t*-test. Normalized pMARCKS/MARCKS expression is derived from an average of 2–3 independent experiments for each cell line. The GNAQ/GNA11-mutant Mel270 and OMM1 cells are highlighted by the crossed, circle symbol. The WT Mel285 and Mel290 UM cells are highlighted in orange. ns not significant.

UM cells were treated with vehicle control or the PKC inhibitors IDE196 and AEB071 and protein changes were monitored 24 h post treatment by western immunoblotting. The selected doses of IDE196 and AEB071 were previously shown to induce UM cell cycle arrest and cell death [19–21]. We examined canonical downstream PKC effector proteins (MARCKS) and PKC-regulated signaling cascades, including the MAPK (pathway markers: ERK, DUSP6, S6), phosphatidylinositol-3-kinase/protein kinase B (PI3K/AKT; pathway markers: AKT, S6) and Yes-associated protein 1/WW domain-containing transcription regulator 1 (YAP/TAZ; pathway marker: YAP) pathways (Figs. 1A and S1A). As expected, inhibition of PKC suppressed the phosphorylation of the PKC effector protein MARCKS in all UM cell lines regardless of their GNAQ/GNA11 mutational status (Figs. 1B and S1B). Both PKC inhibitors showed similar repression of MARCKS phosphorylation, although the two WT UM cell lines showed a greater degree of MARCKS inhibition in response to the AEB071 inhibitor compared to IDE196 (Fig. 1C).

**Fig. 2:**
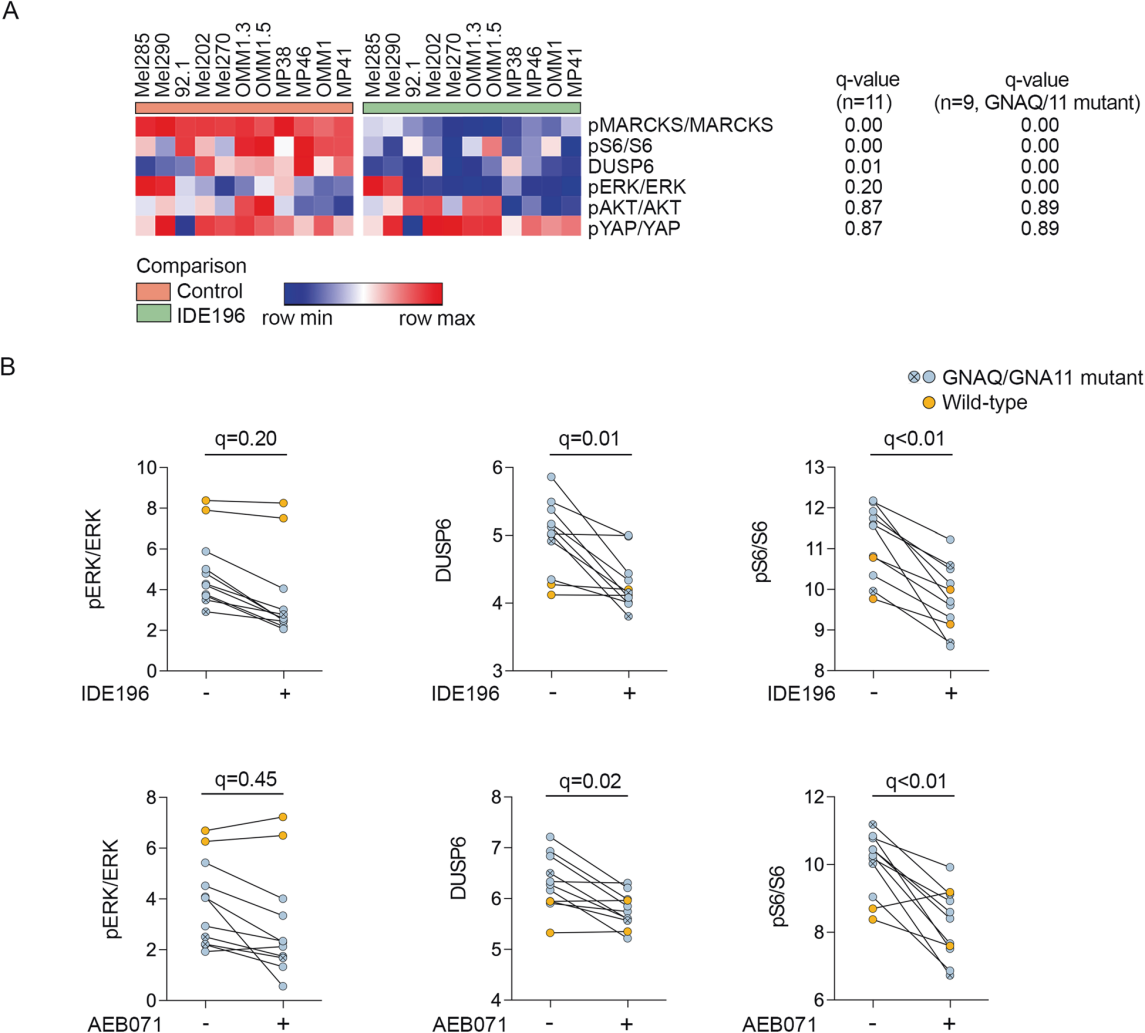
Expression of effector proteins in UM cell lines in response to PKC inhibitors (IDE196 and AEB071). **A** Heatmap showing comparison of the indicated normalized proteins in control-treated vs IDE196-treated (24 h) UM cell lines. The false discovery (FDR) adjusted *p*-values (q-value) are shown for the comparison of control and treated UM cell lines, *n* = 11 and *GNAQ/*GNA11-mutant UM cell lines, *n* = 9, using the t-test within the Morpheus web-based tool (https://software.broadinstitute.org/morpheus/). **B** Normalized expression of the indicated proteins pre- and post- 24 h treatment with 5 µM IDE196 or 5 µM AEB071. The false discovery rate (FDR) adjusted *p*-values (*q*-value) are shown for the comparison of control and treated UM cell lines, *n* = 11 using the *t*-test within the Morpheus web-based tool (https://software.broadinstitute.org/morpheus/).

**Fig. 3:**
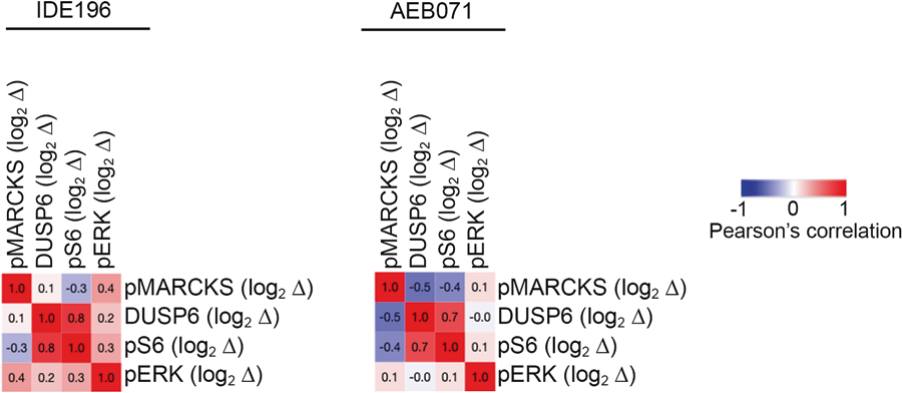
Response of MAPK effector proteins to PKC inhibition. Similarity matrix showing no significant correlation between PKC-inhibitor induced changes (log_2_ fold changes, log_2_ ∆) in pMARCKS and the MAPK signaling markers DUSP6, pS6, and pERK. Pearson correlation coefficients are shown and were determined using the *t*-test within the Morpheus web-based tool (https://software.broadinstitute.org/morpheus/). All UM cell lines (*n* = 11) were included in these analyses.

Two other effector proteins, pS6 and DUSP6 were also significantly downregulated (false discovery rate (FDR)-adjusted *p*-value (*q*) < 0.05) in response to PKC inhibition in the UM cell lines (Figs. 2A and S1C). We noted, however, that the WT UM showed minimal changes in the accumulation of pERK, pS6 and DUSP6 in response to 24 h of PKC inhibitor treatment (Fig. 2B), and when the WT UM were excluded from these analyses, ERK phosphorylation was also shown to be markedly inhibited in the GNAQ/GNA11-mutant UM cell lines (*n* = 9; *q*-value < 0.1; Figs. 2A and S1C). Importantly, the level of PKC inhibitor-induced decreases in MARCKS phosphorylation (i.e log_2_ fold change in MARCKS phosphorylation) did not correlate with the degree of MAPK inhibition, as measured by the PKC inhibitor changes in pERK, pS6, and DUSP6, in the 11 UM cell lines (Fig. 3).

Finally, PKC inhibitor treatment did not consistently affect the phosphorylation of AKT or YAP in these UM cell models (Figs. 2A and S1C). Collectively, these data indicate that PKC inhibition was effective in downregulating MARCKS activation in all UM cell models, but this did not suppress PI3K/AKT or YAP/TAZ signaling and only inhibited the MAPK pathway in the GNAQ/GNA11-mutant UM cells.

### PKC inhibition promotes cell cycle inhibition but not cell death in most GNAQ/GNA11-mutant cell lines

Considering that MARCKS phosphorylation was potently and uniformly inhibited in response to PKC inhibition, we examined the functional consequences of this signaling change. The UM cell lines were exposed to two PKC inhibitor concentrations and cell cycle distribution and viability were analysed 72 h post treatment. In most cell lines the response of UM cell lines to intermediate (1 µM IDE196 or 2 µM AEB071) and high dose (5 µM each) PKC inhibitors was equivalent (Table S1).

**Fig. 4:**
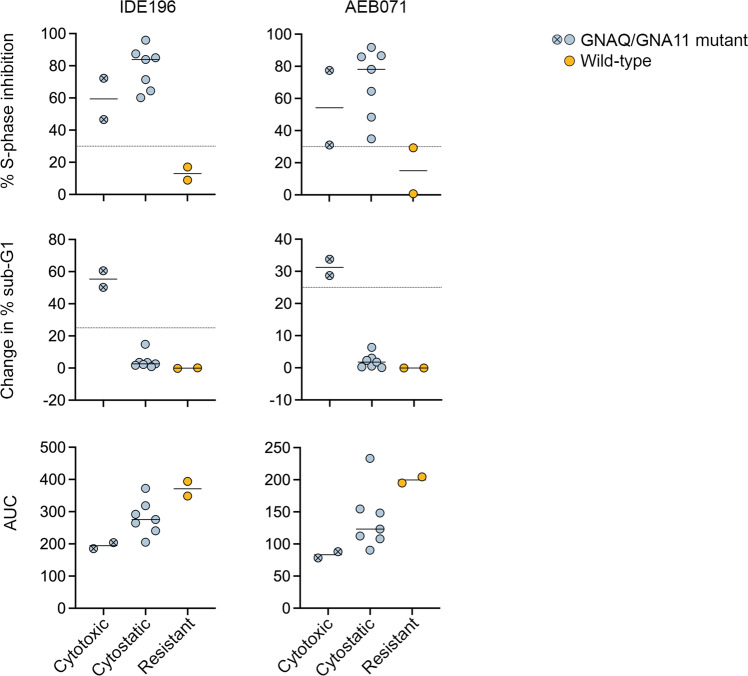
Functional effects of PKC inhibition in UM cell lines. Percent S-phase inhibition (top), change in sub-G1 (middle) and area under dose-response curves (AUC, bottom) are shown for all UM cell lines treated for 72 h with 5 µM IDE196 or 5 µM AEB071. The UM are grouped according to three response patterns - cytotoxic, cytostatic, and resistant. The average of three independent experiments are shown for each cell line. The GNAQ/GNA11-mutant Mel270 and OMM1 cells are highlighted by the crossed, circle symbol. The WT Mel285 and Mel290 UM cells are highlighted in orange.

Analysis of PKC inhibitor responses revealed three distinct UM response patterns – cytotoxic (defined as >25% in sub G1 content), cytostatic (defined as ≥30% S-phase inhibition, at both PKC inhibitor concentrations, without evidence of cell death) and resistant (defined as minor/inconsistent changes in cell cycle distribution with no evidence of cell death in response to PKC inhibition). PKC inhibition was cytotoxic in only the GNAQ-mutant Mel270 and GNA11-mutant OMM1 cell lines. These two cell lines showed substantial cell death (defined as >25% in sub G1 content), particularly in response to IDE196 (> 50% change in sub G1; Fig. 4). Another seven UM cells lines displayed a cytostatic response after treatment with PKC inhibitors. These cells displayed ≥ 30% S-phase inhibition, at both PKC inhibitor concentrations, without evidence of cell death. Cytostatic UM cell lines included the 92.1, Mel202, OMM1.3, OMM1.5, MP38, MP41, and MP46. The WT Mel290 and Mel285 cell lines were resistant to IDE196 and AEB071 (Fig. 4 and Table S1). It is worth noting that although the Mel285 cells showed some evidence of S-phase inhibition in response to AEB071 (almost 30% S-phase inhibition), this was not consistent between the intermediate and high AEB071 concentrations (Table S1).

These three PKC inhibitor response patterns were also consistent with drug-dose cell viability (MTT response) assays, with the two most sensitive UM cell lines, Mel270 and OMM1, displaying the lowest AUC values for both AEB071 and IDE196 (Fig. 4).

### PKC-regulated MAPK activity regulates UM proliferation

**Fig. 5:**
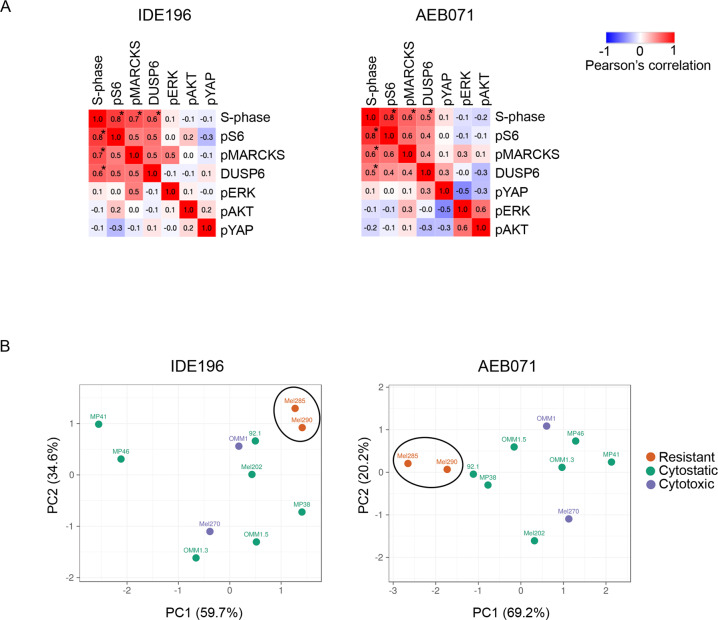
UM proliferation reflects MAPK activity. **A** Similarity matrix showing Pearson correlation coefficient between % S-phase cell content pre and post 72 h PKC inhibitor treatment and the indicated normalized effector proteins (data derived from western immunoblotting). Pearson correlation coefficients determined the Morpheus web-based tool. All UM cell lines were included in these analyses, and asterisks indicate *p*-value < 0.05. **B** PCA plot derived from the log_2_ fold change values of DUSP6, pMARCKS, and pS6 protein levels differentiated cytotoxic/cytostatic UM cell lines from resistant UM cell lines, but did not differentiate between the cytotoxic and cytostatic UM cell lines. ClustVis was used to generate PCA plots.

The number of PKC inhibitor resistant and sensitive UM cell lines were too small to allow for meaningful statistical comparisons between the resistant, cytostatic and cytotoxic UM PKC inhibitor response groups. It was evident, however, that the cytotoxic Mel270 and OMM1 cell lines did not show the most pronounced inhibition of pMARCKS, pS6, DUSP6, or pERK (Figs. 1, 2, and S1). Therefore, we examined the relationship between UM proliferation (i.e % S-phase) and survival (% sub G1) and signaling protein markers. As shown in Fig. 5A, accumulation of pMARCKS, pS6, and DUSP6 with and without PKC inhibitor treatment was significantly correlated with S-phase cell content (but not sub G1; data not shown) in the UM cells. We also found that PKC inhibitor-induced changes in pMARCKS, DUSP6 and pS6 differentiated the cytotoxic and cytostatic UM cell lines from the resistant cell models using a Principal Component Analysis (PCA) plot, although this protein signature was unable to differentiate between the cytotoxic and cytostatic UM cell lines (Fig. 5B).

These data indicate that PKC regulates MAPK activity in GNAQ/GNA11 mutant, but not wild type, UM cell lines, and that MAPK activity is strongly associated with UM cell proliferation, but not cell survival.

### PKC inhibitor monotherapy is not sufficient to suppress multiple pathways active in UM

To expand our analysis on PKC inhibitor-induced signaling changes in UM, we examined the transcriptome profiles of five uveal melanoma cell lines treated with vehicle control (dimethylsulfoxide; DMSO) or 5 µM IDE196 for 8 h, 16 h and 24 h. These UM cells included the Mel270 (cytotoxic response), three cell lines showing cytostatic responses (OMM1.3, MP38, and MP46) and the resistant Mel285 cell line.

Geneset enrichment analysis using GSEA in PreRanked mode and the Hallmark gene set with additional signatures indicative of MAPK and YAP activity, was used to identify gene signatures that were differentially expressed between control versus PKC inhibitor-treated uveal cell models. For each cell line the differentially expressed genes were ranked and selected based on an FDR-adjusted *p*-value < 0.05.

**Fig. 6:**
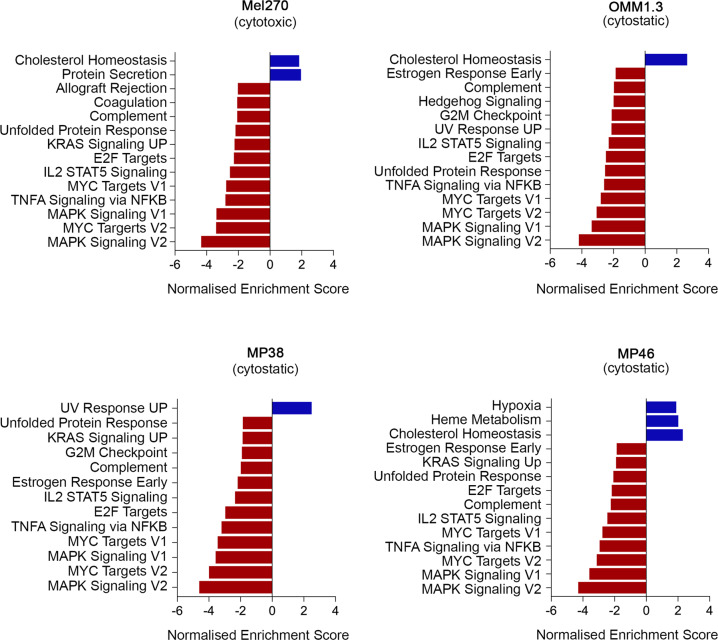
PKC inhibition represses transcriptome genesets indicative of proliferation and MAPK signaling in responsive and sensitive UM. Geneset enrichment analyses was performed using GSEA PreRanked mode and the Hallmark gene set with additional signatures indicative of MAPK and YAP activity, was used to identify gene signatures that were differentially expressed between control versus IDE196 (5 µM) inhibitor-treated (8 h, 16 h, and 24 h treatment time points were combined) UM cells. For each cell line the differentially expressed genes were ranked and selected based on an FDR-adjusted *p*-value < 0.05. The complete geneset enrichment analysis data are shown in Tables S2–S5. PKC inhibitor treatment of the resistant Mel285 UM cell line did not produce any significant changes in transcriptome signatures.

**Fig. 7:**
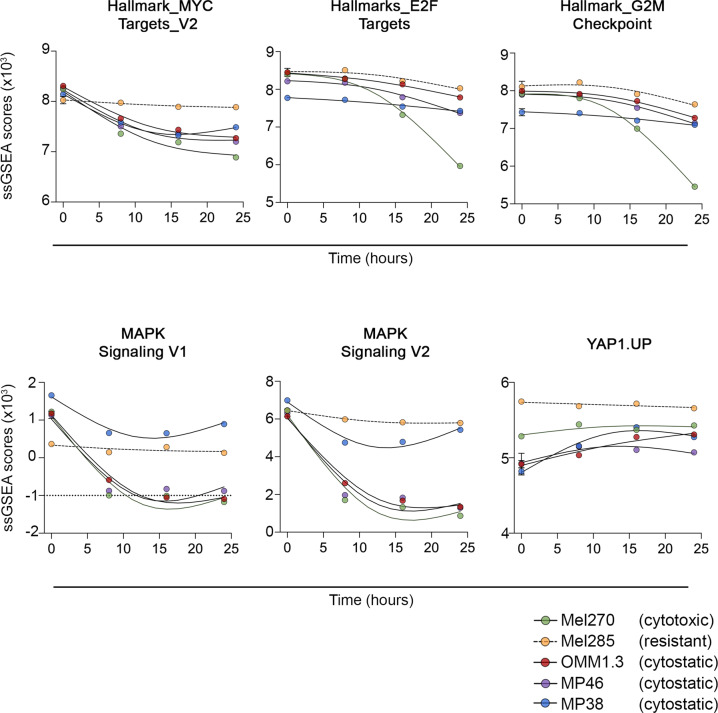
Temporal analysis of proliferative signatures using ssGSEA of 5 UM cell lines. Single-sample gene set enrichment analysis (ssGSEA) scores for the indicated genesets are plotted over PKC inhibitor treatment time.

The cytotoxic Mel270 cell line showed consistent loss of several signatures indicative of cell cycle inhibition, including the Hallmark_E2F Targets and Hallmark MYC Targets_V1 & V2 gene sets (Fig. 6 and Table S2). Temporal analysis of proliferative signatures using ssGSEA confirmed that these proliferation genesets were inhibited as early as 8 h after PKC inhibitor treatment in the Mel270 cell line with further loss observed 24 h of PKC inhibitor treatment (Fig. 7). PKC inhibition was also associated with reduced MAPK, mammalian target of rapamycin C (mTORC) and ß-catenin signaling activity in the Mel270 cell line (Table S2).

ssGSEA analysis confirmed that MAPK activity loss (MAPK Signaling V1, V2) was pronounced 8 h post PKC inhibitor treatment and plateaued by 16 h of treatment (Fig. 7). In contrast, although the mTORC1 and WNT-ß-catenin genesets were reduced in the GSEA preRanked analysis in response to PKC inhibitor treatment (Table S2), ssGSEA temporal analysis confirmed that these changes were minimal and not always consistent during the PKC inhibitor treatment time course (Fig. S2). Importantly, we observed no reduction in the YAP or the Janus kinase (JAK) signaling cascades, which are established downstream GNAQ effector pathways (Table S2).

The three cytostatic UM cell models (MP38, MP46, and OMM1.3) also showed a consistent decrease in proliferative gene sets (Fig. 6 and Tables S2–S5). Temporal analysis of these proliferative signatures revealed that the loss of proliferative signatures was not as pronounced in the cytostatic uveal cell lines when compared to the cytotoxic Mel270 cell model (Fig. 7). Nevertheless, MAPK signaling was rapidly and potently inhibited in the OMM1.3 and MP46 cell lines, whereas MAPK signaling loss was not as pronounced in the MP38 cell model and there was evidence of MAPK signaling recovery at 24 h of PKC inhibitor treatment (Fig. 7).

There was no significantly differentially expressed gene signatures in the resistant Mel285 cell line (control vs PKC inhibitor treatment groups) even though the ranking used for selecting genes in this cell model was increased to an FDR-adjusted p value <0.2.

It is also worth noting that few transcriptome gene signatures were upregulated in response to PKC inhibition, although the cholesterol homeostasis was increased in Mel270, OMM1.3 and MP46 cell models (Fig. 6). PKC is a major regulator of cholesterol and lipid metabolism [22].

We also examined the hallmark signature profile of the UM cell lines at baseline (i.e control-treated). Predictably there was substantial variation in the ssGSEA scores of signaling pathways across these UM cell models. For instance, the PKC inhibitor-resistant Mel285 UM had the highest ssGSEA scores for epithelial-mesenchymal transition, transforming growth factor ß (TGFß) signaling, tumor necrosis factor α (TNFα) signaling, and kirsten rat sarcoma virus (KRAS) signaling (Fig. S3A). These signatures are consistent with the classification of Mel285 cells as undifferentiated, based on four melanoma transcriptome subtypes (undifferentiated, neural-crest like, transitory and undifferentiated) recently defined by Tsoi et al. [23]. In contrast the cytotoxic Mel270 UM cell showed the lowest expression for many hallmark gene sets including epithelial-mesenchymal transition, interleukin 6-Janus kinase-signal transducer and activator of transcription 3 (IL6-JAK-STAT3), Hedgehog and KRAS signaling. Mel270 and OMM1.3 models were classified as melanocytic and PKC inhibitor treatment over 24 h did not alter this differentiation status and the MP38 uveal melanoma cell line remained transitory in the presence of PKC inhibition (Fig. S3B).

## Discussion

This study comprehensively examined the response of a large panel of UM cell lines to two clinically developed PKC inhibitors. We confirm that both AEB071 and IDE196 consistently inhibited the phosphorylation of MARCKS, an immediate downstream effector of PKC. Critically, the inhibition of MARCKS was not sufficient to predict the response of UM to PKC inhibition. This was particularly evident for the WT UM cell lines, Mel290 and Mel285. Both these UM cells responded to PKC inhibition with potent suppression of MARCKS phosphorylation, although this did not result in the downregulation of established UM oncogenic and PKC-regulated pathways, including MAPK [15, 24] and PI3K signaling [25]. The WT UM also continued proliferating in response to PKC inhibitor treatment, and thus MAPK activity, cell proliferation and survival in WT UM models was independent of PKC activity.

The nine GNAQ/GNA11-mutant UM cell lines also responded to PKC inhibition with potent downregulation of MARCKS phosphorylation. In these mutant UM cell lines suppression of MARCKS activity was associated with the downregulation of MAPK signaling at both transcript and protein levels. However, the degree of MARCKS inhibition did not reflect the level of MAPK suppression or cell responses to PKC inhibition. For instance, only the Mel270 and OMM1 UM cells showed cell death in response to PKC inhibitor monotherapy, but these two cell lines did not show the most potent suppression of MARCKS or MAPK signaling. In fact, the differences in UM cell responses to PKC inhibition were not attributable to the degree or timing of pMARCKS, pS6 or DUSP6 inhibition, or the transcriptional inhibition of additional signaling cascades. Instead, UM sensitivity to PKC inhibition is likely to reflect the activity of alternate survival and proliferation pathways, rather than efficacy of PKC inhibitors to downregulate MARCKS and MAPK signaling. This may include pathways that alter the differentiation status of UM cell models. For instance, the PKC inhibitor resistant Mel285 UM cells expressed elevated TGFß signaling and displayed features of treatment resistant undifferentiated melanoma [23].

Our data are consistent with previous literature describing UM responses to PKC inhibition. In particular, AEB071 consistently inhibited the phosphorylation of MARCKS in six UM cell lines, regardless of the cell genotype. However only GNAQ/11-mutant UM cell lines responded to AEB071 by suppressing MAPK activity and undergoing dose-dependent proliferative inhibition [26].

Our work is also consistent with clinical trial data showing limited anti-tumor effects of therapies targeting single oncogenic pathways in UM. For instance, phase I clinical trial with MEK inhibitors TAK-733 and trametinib have shown that UM are poorly responsive [27, 28]. The phase 3 clinical trial comparing the MEK inhibitor selumetinib or placebo in combination with dacarbazine failed to meet primary and secondary endpoints on PFS and OS [29]. Similarly, only 4/153 (3%) and 6/66 (9%) metastatic UM had a partial response to PKC inhibitor monotherapy, AEB071 or IDE196, respectively [18, 30]. Despite promising pre-clinical studies using various combination therapies, PKC inhibitor monotherapy failed to translate in clinical trials. Selumetinib combined with AKT inhibitor MK2206 showed synergistic effect in GNAQ-mutant UM cell lines and decreased tumor growth in xenograft mouse models [31] however in a phase 2 clinical trial, efficacy of trametinib with or without AKT inhibitor GSK2141795 failed to show any survival benefit with no difference in PFS, and only one PR was observed with ORR of 5% [32]. Similarly, although combination MEK inhibitor (MEK162) and PKC inhibitor (PD0325901) treatment showed synergistic activity in a UM model in vivo [15] a phase 1b/2 clinical trial using MEK plus PKC inhibitors (binemetinib with AEB071) was terminated prior to phase 2 expansion (NCT01801358) due to toxicity. The combination of PKC inhibitor AEB071 with PI3K inhibitor BYL719 showed synergistic effect in pre-clinical models [26] and is currently in a phase 1 clinical trial setting (NCT02273219). IDE196 is also being investigated in combination with small molecule inhibitors of MEK (binimetinib) or mutated anaplastic lymphoma kinase (ALK; crizotinib) in phase 1/2 clinical trials of solid tumors with GNAQ/11 mutations (NCT03947385). Additional novel treatment combinations are also under investigation, and the combination of MEK inhibition with focal adhesion kinase (FAK) inhibitor has shown promising efficacy in pre-clinical models [33]. Finally, new therapies selectively targeting GNAQ/GNA11 have been developed. The structurally similar FR900359 and YM-254890 inhibit oncogenic GNAQ/GNA11 and promote potent cell cycle arrest and apoptosis in uveal melanoma [19, 34, 35]. Combining YM-254890 and MEK inhibitor led to sustained MAPK inhibition and synergistic growth inhibition in vitro and tumor shrinkage in vivo ([36].

Our data confirm that PKC activity is potently suppressed in UM by the clinically available AEB071 and IDE196 PKC inhibitors. PKC inhibition does not inhibit multiple activated Gα downstream pathways however, and the suppression of MAPK activity was not sufficient to promote robust cell death in most UM cell models. Instead, novel combination therapies targeting multiple upstream and downstream signaling nodes will be required to inhibit the complex signaling pathways that contribute to the proliferation and survival of UM.

## Materials and methods

### Uveal melanoma cell lines

A total of 11 cell lines, Mel285, Mel290, 92.1, Mel202, Mel270, OMM1.3, OMM1.5, MP38, MP46, OMM1, and MP41 were included in this study (Table 1). UM cell lines were donated by Professor Elin S. Gray, Edith Cowan University, Western Australia and Professor Bruce R. Ksander, Harvard Medical School, USA and Professor Martine J. Jager, University of Leiden, Netherlands. Cell authentication and profiling of UM cell lines was confirmed using the StemElite ID system (Promega, Madison, WI) and all cells tested negative for mycoplasma (MycoAlert Mycoplasma Detection Kit, Lonza, Basel).

Cell lines were cultured in Roswell Park Memorial Institute-1640 media supplemented with 10 or 20% heat inactivated fetal bovine serum (FBS; Sigma-Aldrich, St. Louis, MO, USA), 4 mM glutamine (Gibco, Thermo Fisher Scientific, Waltham, MA, USA), and 10 mM HEPES (Gibco) and were maintained at 37 °C in 5% CO_2_.

### PKC inhibitors

AEB071 was purchased from Selleckchem (S2791) and prepared as a 10 mM stock solution in DMSO. IDE196 was purchased from Chemgoods (C-1368) and prepared as a 10 mM stock solution in DMSO. All stocks were stored in aliquots at −20 °C. For western blot analysis, 5μM was used for AEB071 and IDE196. For cell cycle analysis, 1 μM and 5 μM for AEB071 and 2 μM and 5 μM for IDE196 were used. For cell viability assay, doses ranging from 0.001 μM to 10 μM for AEB071 and 0.1 μM to 10 μM for IDE196 were used. Transcriptome analysis was performed after treating cells for 24 h with 5 µM IDE196.

### Western blot analysis

UM cell lines were treated with 5μM AEB071 or 5μM IDE196 or 0.1% DMSO for 24 h. Total cellular proteins were extracted at 4 °C using RIPA lysis buffer containing protease inhibitors and phosphatase inhibitors (Roche, Basel, Switzerland). Total proteins (20 µg) were resolved on 10% SDS-PAGE and transferred to Immobilon-P membranes (Millipore). Western blots were probed with the following antibodies: total ERK (1:2000, 4695, Cell Signaling, Danvers, MA), phosphorylated ERK (Tyr204,1:1000, SC-7383, Santa Cruz, Dallas, TX), total AKT (1:1000, 2920, Cell Signaling), phosphorylated AKT (Ser473, 1:500, 3787, Cell Signaling), DUSP6 (1:1000, AB76310, Abcam, Cambridge, United Kingdom), total YAP (1A12, 1:500, 123955, Cell Signaling), phosphorylated YAP (Ser127, 1:2000, 4911, Cell Signaling), total MARCKS (FC141–2C2, Sigma), phosphorylated MARCKS (Ser152/156, 1:1000, 2741S, Cell Signaling), total ribosomal S6 (1:500, 54D2, Cell Signaling), phosphorylated ribosomal S6 (Ser235/236, 1:1000, 2F9, Cell Signaling) overnight at 4 °C. Membranes were washed with tris-buffered saline with 0.05% Tween-20 and then incubated with secondary antibodies IRDye® 800CW Donkey anti-Mouse, IRDye® 800CW Donkey anti-Rabbit, IRDye® 680LT Donkey anti-Mouse, IRDye® 680LT Donkey anti-Rabbit (LI-COR, Lincoln, NE). Membranes were detected on the Odyssey imaging system. pERK, pYAP, pAKT, pMARCKS, pS6 were normalized relative to their respective total protein levels and DUSP6 was normalized to the REVERT total protein stain (LI-COR). Normalized protein data were log_2_ transformed (multiplied by a factor of 1000) and independent experiments averaged.

### Cell cycle analysis

UM cells were treated with various inhibitors for 72 h and floating and adhered cells collected for cell cycle and apoptosis analysis as previously described [37]. DNA content from 10,000 cells was analysed using the ModFIT LT 5.0 software (Verity Software House, Topsham, ME) and numbers of dead cells (sub G1 phase) were determined using the FACSDiVa software (Becton Dickinson, Franklin Lakes, NJ). The percentage of S phase inhibition was calculated as [((percentage of S phase in the control-treated cells – percentage of S phase in drug-treated cells)/(percentage of S phase in the control-treated cells)) × 100]. Change in percentage sub G1 was calculated relative to the control-treated cells (% sub G1 in treated cells – % sub G1 in control-treated cells).

### Cell viability assays

UM cells were seeded in 96-well plates at 2.0 × 10^3^ cells per well and incubated overnight in RPMI-1640. Cells were allowed to adhere for 24 h before treatment. Media was then removed and UM cells were exposed to increasing concentrations of PKC inhibitor for 72 h; AEB071 (0, 0.1, 0.25, 0.5, 1, 2, 4, 5, 7.5, and 10 μM) and IDE196 (0, 0.001, 0.005, 0.01, 0.05, 0.1, 0.5, 1, 5, and 10 μM). Cell viability was measured using the Cell titre 96 Aqueous One Solution Cell Proliferation Assay (Promega, G358B). The absorbance density was acquired on a PHERASTAR F5 microplate reader (BMG LABTECH, Ortenberg, Germany) at a wavelength of 490 nm. Cell viability was calculated as a percentage normalized to controls after background subtraction. A minimum of three independent viability assays, each conducted in triplicate was performed for each cell line. We report area under the does response curve (AUC) for these analyses as the UM cell models showed variable maximal growth inhibition [38] and IC_50_ values did not accurately discriminate between these response curves. Cell viability measurements were fitted using GraphPad Prism software version 9.0 (GraphPad, San Diego, CA).

### RNA isolation and sequencing

Total RNA was isolated from uveal melanoma cell lines using the RNeasy Kit (Qiagen, Hilden, Germany). cDNA synthesis and library construction were performed using the TruSeq RNA Library Prep Kit (Illumina, San Diego, CA, USA) and paired-end 150 bp sequencing, with each sample yielding 40–50 million reads. Sequencing was performed on the Illumina NovaSeq platform at the Australian Genome Research Facility.

### RNA-sequence data processing

RNA-sequence data processing was kindly performed by Dr Dario Strebnac, University of Sydney. Trimming of Illumina TruSeq paired-end sequencing adapter sequences and bases with a quality of <20 from each end was done using cutadapt [39]. Reads <50 bases long after trimming were discarded from subsequent analysis. The filtered reads were mapped to reference genome hg38 using STAR with 10% mismatches allowed. Reads that mapped equally to more than one genomic location were discarded. Reads were imported into R with GenomicAlignments read GAlignmentPairs function. StrandMode was set to 2. GENCODE Genes version 26 was used as the gene reference database. Counting of reads overlapping with exonic regions of each gene was done with the countOverlaps function from GenomicAlignments.

RNA count data were normalized using trimmed mean of M-values (TMM) and transformed with voom to log2-counts per million with associated precision weights [40]. To obtain abundance values corrected for transcript lengths, as required by the single-sample gene set enrichment analysis (ssGSEA) [41], RSEM (RNA-Seq by Expectation Maximization) was used to derive the fragments per kilobase of transcript per million mapped reads (FPKM) estimates using GENCODE Genes version 26 as the reference transcript database.

### Differentially expressed gene analysis

Differentially expressed genes between control and PKC inhibitor treatment groups were determined using TMM-voom normalized log_2_ counts [42] and the moderated t-tests (limma package version 3.48.1 in R/Bioconductor) [40]. The control-treated cells (including the 8 h, 16 h and 24 h time points) were compared to PKC inhibitor-treated cell lines (including the 8 h, 16 h and 24 h treatment time points). A false discovery rate (FDR) *q* < 0.05 was used to generate the ranked gene list for each cell line. Enrichment scores were calculated from the ranked lists using gene set enrichment analysis in pre-ranked mode (GSEA pre-ranked version 7.2.4) [43] provided by GenePattern (https://cloud.genepattern.org/) [44]. Two established transcriptional signatures of MAPK activation (MAPK Signaling V1 [45], MAPK Signaling V2 [46]) and the YAP1.UP geneset (MSigDb signature within the C6: oncogenic signature collection) were included as part of the h.all.v7.4 hallmark gene set.

Transcriptome analysis of log2 (FPKM + 1) count data was performed using single-sample gene set enrichment analysis (ssGSEA version 10.09) [41] provided by GenePattern (https://cloud.genepattern.org/) [44].

### Statistical analysis

Analysis was performed using the GraphPad Prism software version 9.0 (GraphPad Software, San Diego, CA). was used. Figure legends specify the statistical analysis used and define error bars. If the assumption of normality distribution was not met, nonparametric tests were used. Equality of variances across unpaired samples was confirmed using an F-test as part of unpaired t-test analyses. Comparisons between control and PKC inhibitor treated UM cell lines were calculated using the Marker Selection tool, the Pearson’s correlation coefficient in the nearest neighbor and Similarity Matrix algorithms within the Morpheus web-based tool (https://software.Broadinstitute.org/morpheus/). ClustVis was used for principal component analysis [47].

## Supplementary information

Supplementary Tables

Supplementary Figures

## Data Availability

The RNAseq data is deposited in the Sequence Read Archive (SRA) BioProject PRJNA800601. All other data is available within the Article, Supplementary Information or available from the authors upon reasonable request.
