# Supplementary material for: Protein kinase inhibitor responses in uveal melanoma reflects a diminished dependency on PKC-MAPK signaling
Source: Cancer Gene Ther. 2022 Mar 29;29(10):1384–93. doi: 10.1038/s41417-022-00457-2 (PMC9576594; doi:10.1038/s41417-022-00457-2)
Supplement: Supplementary file 1 — Supplementary Tables [file 41417_2022_457_MOESM1_ESM.pdf]

Table S1 Cell cycle distribution data of uveal melanoma cells treated with protein kinase C (PKC) inhibitors IDE196 or AEB071

			5µM IDE196		1µM IDE196		5µM AEB071		2µM AEB071	
**Cell Lines**	**PKC inhibitor**	**Response type**	**% S phase**	**% Sub G1**	**% S phase**	**% Sub G1**	**% S phase**	**% Sub G1**	**% S phase**	**% Sub G1**
Mel285	-	Resistant	14.2	1.1	14.2	1.1	11.7	1.7	11.7	1.7
Mel285	+	Resistant	12.9	0.9	13.9	1.0	8.3	1.7	10.1	1.4
Mel290	-	Resistant	7.4	0.0	7.4	0.0	7.2	0.1	7.2	0.1
Mel290	+	Resistant	6.1	0.1	6.4	0.1	2.1	0.0	5.4	0.1
92.1	-	Cytostatic	29.7	0.6	29.7	0.6	30.6	0.8	30.6	0.8
92.1	+	Cytostatic	1.2	15.4	3.9	3.8	2.5	1.3	4.3	4.1
Mel202	-	Cytostatic	27.9	0.2	27.9	0.2	31.4	0.1	31.4	0.1
Mel202	+	Cytostatic	9.9	3.6	10.7	1.4	11.2	6.5	9.7	2.8
Mel270	-	Cyototoxic	19.1	0.7	19.1	0.7	20.5	0.5	20.5	0.5
Mel270	+	Cyototoxic	5.3	61.2	4.2	65.7	4.6	29.2	5.1	30.6
OMM1.3	-	Cytostatic	28.1	0.2	28.1	0.2	32.7	0.3	32.7	0.3
OMM1.3	+	Cytostatic	4.5	3.8	4.5	8.6	4.6	3.3	9.0	3.0
OMM1.5	-	Cytostatic	25.5	2.0	25.5	2.0	27.0	1.3	27.0	1.3
OMM1.5	+	Cytostatic	3.8	4.6	3.8	4.6	5.9	3.6	5.5	3.5
MP38	-	Cytostatic	11.1	0.2	11.1	0.2	10.8	0.4	10.8	0.4
MP38	+	Cytostatic	3.2	1.1	5.3	0.6	5.6	0.7	9.1	0.5
MP46	-	Cytostatic	23.8	7.8	23.8	7.8	22.5	3.3	22.5	3.2
MP46	+	Cytostatic	9.5	9.5	11.9	9.7	14.7	5.1	14.9	5.9
OMM1	-	Cyototoxic	32.1	1.8	32.1	1.8	30.3	1.1	30.3	1.1
OMM1	+	Cyototoxic	17.2	52.0	24.9	38.4	20.9	34.9	27.8	30.3
MP41	-	Cytostatic	24.6	1.0	24.6	1.0	24.7	0.8	24.7	0.8
MP41	+	Cytostatic	3.1	3.2	4.4	2.5	3.3	0.8	5.0	0.9

Data are the average of at least three biological resplicates

Table S2 Preranked geneset enrichment analysis comparing PKC inhibitor- versus control-treated Mel270 uveal melanoma cell line

NAME	SIZE*	ES	NES	NOM p-val	FDR q-val
HALLMARK_PROTEIN_SECRETION	41	0.35552156	1.9543794	0.003125	0.03183081
HALLMARK_CHOLESTEROL_HOMEOSTASIS	39	0.34009954	1.8353789	0.003378379	0.04084871
HALLMARK_HEME_METABOLISM	80	0.2705555	1.7902172	0.007843138	0.0364024
HALLMARK_MYOGENESIS	58	0.26834813	1.6657465	0.017985612	0.06430768
HALLMARK_MITOTIC_SPINDLE	73	0.22429891	1.5008874	0.017777778	0.14021592
HALLMARK_ADIPOGENESIS	88	0.19336279	1.3648218	0.061320756	0.23424698
HALLMARK_BILE_ACID_METABOLISM	29	0.25977308	1.2720866	0.15542522	0.32259548
YAP1_UP	14	0.32472456	1.2230203	0.21938775	0.35354346
HALLMARK_INTERFERON_ALPHA_RESPONSE	33	0.23495534	1.2209482	0.19020173	0.31721726
HALLMARK_PEROXISOME	40	0.2041939	1.1388118	0.2861635	0.40473446
HALLMARK_FATTY_ACID_METABOLISM	72	0.17167652	1.1230505	0.27659574	0.39097106
HALLMARK_PI3K_AKT_MTOR_SIGNALING	49	0.1874609	1.0959275	0.3028169	0.40036893
HALLMARK_XENOBIOTIC_METABOLISM	71	0.16025962	1.0656121	0.3231441	0.41639012
HALLMARK_APICAL_JUNCTION	67	0.15530641	0.9956903	0.48062015	0.5002682
HALLMARK_OXIDATIVE_PHOSPHORYLATION	90	0.12365571	0.8770987	0.6492891	0.65099233
MAPK Signaling V2	45	-0.9043531	-4.3440814	0	0
HALLMARK_MYC_TARGETS_V2	51	-0.6880464	-3.4035542	0	0
MAPK Signaling V1	27	-0.796536	-3.386963	0	0
HALLMARK_TNFA_SIGNALING_VIA_NFKB	94	-0.4968598	-2.8191597	0	0
HALLMARK_MYC_TARGETS_V1	126	-0.4705812	-2.7702599	0	0
HALLMARK_IL2_STAT5_SIGNALING	90	-0.4481024	-2.5309303	0	0
HALLMARK_E2F_TARGETS	70	-0.4338211	-2.2805126	0	3.95E-04
HALLMARK_KRAS_SIGNALING_UP	57	-0.4413556	-2.2384841	0	4.74E-04
HALLMARK_UNFOLDED_PROTEIN_RESPONSE	69	-0.4052557	-2.1830726	0	8.52E-04
HALLMARK_COMPLEMENT	60	-0.4022088	-2.075302	0	0.00158165
HALLMARK_COAGULATION	42	-0.4446047	-2.072712	0	0.00143787
HALLMARK_ALLOGRAFT_REJECTION	44	-0.4292351	-2.0350122	0	0.00243398
HALLMARK_GLYCOLYSIS	82	-0.3634427	-1.9952645	0	0.00327154
HALLMARK_UV_RESPONSE_UP	74	-0.3539345	-1.8797234	0.00257732	0.00755535
HALLMARK_ANDROGEN_RESPONSE	56	-0.3689057	-1.8480057	0.004081633	0.01008498
HALLMARK_INFLAMMATORY_RESPONSE	56	-0.3568153	-1.7883152	0.004065041	0.0157092
HALLMARK_ESTROGEN_RESPONSE_EARLY	86	-0.3006454	-1.6494657	0.008805032	0.04173814
HALLMARK_MTORC1_SIGNALING	115	-0.2757017	-1.6464698	0.007462686	0.04017502
HALLMARK_WNT_BETA_CATENIN_SIGNALING	19	-0.438736	-1.6338125	0.037459284	0.04134335
HALLMARK_REACTIVE_OXYGEN_SPECIES_PATHWAY	24	-0.4008331	-1.6061797	0.04105572	0.04666503
HALLMARK_APOPTOSIS	68	-0.3038231	-1.5893749	0.014304291	0.04928973
HALLMARK_HYPOXIA	95	-0.2766756	-1.5646012	0.03345725	0.05512825
HALLMARK_G2M_CHECKPOINT	76	-0.2921255	-1.5477916	0.025435073	0.0585655
HALLMARK_P53_PATHWAY	88	-0.2677643	-1.4733243	0.04928664	0.08987591
HALLMARK_NOTCH_SIGNALING	11	-0.4587637	-1.4175186	0.109350234	0.11798614
HALLMARK_TGF_BETA_SIGNALING	27	-0.327182	-1.3615142	0.1337386	0.15183696
HALLMARK_UV_RESPONSE_DN	80	-0.2430972	-1.3319138	0.11973684	0.16927467
HALLMARK_IL6_JAK_STAT3_SIGNALING	23	-0.3229279	-1.2647998	0.20276497	0.22358574
HALLMARK_INTERFERON_GAMMA_RESPONSE	62	-0.242559	-1.2576683	0.18741633	0.22294049
HALLMARK_EPITHELIAL_MESENCHYMAL_TRANSITION	75	-0.216455	-1.1631231	0.25096524	0.32300726
HALLMARK_ESTROGEN_RESPONSE_LATE	73	-0.2003967	-1.0917333	0.34031415	0.405842
HALLMARK_KRAS_SIGNALING_DN	38	-0.2176059	-0.9832861	0.4848485	0.5499188
HALLMARK_ANGIOGENESIS	11	-0.3040385	-0.9702917	0.47682118	0.55255866
HALLMARK_DNA_REPAIR	52	-0.160704	-0.8132291	0.7239513	0.76923996
HALLMARK_APICAL_SURFACE	13	-0.2344968	-0.7758745	0.74957985	0.79796237
HALLMARK_SPERMATOGENESIS	25	-0.1796924	-0.7312603	0.8021148	0.82757276

ES, Enrichment score
NES, Normalised enrichment score
NOM p-value, Nominal p value
FDR q-value, False discovery rate
*Number of genes in the the gene set after filtering genes not in the expression dataset

Table S3 Preranked geneset enrichment analysis comparing PKC inhibitor- versus control-treated OMM1.3 uveal melanoma cell line

NAME	SIZE*	ES	NES	NOM p-val	FDR q-val
HALLMARK_CHOLESTEROL_HOMEOSTASIS	28	0.5105943	2.656983	0	0
HALLMARK_APICAL_JUNCTION	54	0.27038708	1.7495683	0.004830918	0.09268095
HALLMARK_HEME_METABOLISM	68	0.24924944	1.7013255	0.011111111	0.07974342
HALLMARK_BILE_ACID_METABOLISM	23	0.34493575	1.7000505	0.01618123	0.06073983
HALLMARK_INTERFERON_ALPHA_RESPONSE	31	0.2783709	1.5035703	0.05882353	0.16275918
HALLMARK_KRAS_SIGNALING_DN	29	0.27381226	1.4410456	0.06271777	0.18355565
HALLMARK_PEROXISOME	37	0.25181556	1.4158772	0.06909091	0.17914084
HALLMARK_EPITHELIAL_MESENCHYMAL_TRANSITION	62	0.19217308	1.2894152	0.14220184	0.2869846
HALLMARK_XENOBIOTIC_METABOLISM	66	0.18341698	1.2100248	0.23451327	0.36291468
HALLMARK_MYOGENESIS	55	0.18464121	1.1774857	0.21719457	0.37799564
HALLMARK_FATTY_ACID_METABOLISM	55	0.18078801	1.1439279	0.29577464	0.39240074
HALLMARK_MITOTIC_SPINDLE	67	0.16432121	1.1369663	0.23671497	0.37140548
YAP1_UP	14	0.22673638	0.8803182	0.59052926	0.78906345
HALLMARK_IL6_JAK_STAT3_SIGNALING	17	0.19380076	0.81587285	0.6727829	0.8369996
HALLMARK_PI3K_AKT_MTOR_SIGNALING	42	0.10995829	0.6648202	0.87053573	0.96653694
HALLMARK_INTERFERON_GAMMA_RESPONSE	56	0.09516167	0.62018055	0.94849783	0.93949103
MAPK Signaling V2	45	-0.8697477	-4.192273	0	0
MAPK Signaling V1	24	-0.8344674	-3.375727	0	0
HALLMARK_MYC_TARGETS_V2	52	-0.6153655	-3.0690699	0	0
HALLMARK_MYC_TARGETS_V1	135	-0.4639472	-2.801597	0	0
HALLMARK_TNFA_SIGNALING_VIA_NFKB	96	-0.4512871	-2.5900338	0	0
HALLMARK_UNFOLDED_PROTEIN_RESPONSE	68	-0.4734384	-2.531077	0	0
HALLMARK_E2F_TARGETS	96	-0.4435486	-2.480278	0	0
HALLMARK_IL2_STAT5_SIGNALING	78	-0.4244774	-2.302123	0	4.38E-04
HALLMARK_UV_RESPONSE_UP	65	-0.4013015	-2.1261473	0	0.00128943
HALLMARK_G2M_CHECKPOINT	85	-0.3792525	-2.1156983	0.001199041	0.00142759
HALLMARK_HEDGEHOG_SIGNALING	14	-0.5910172	-1.9884993	0.002902758	0.00399712
HALLMARK_COMPLEMENT	54	-0.3890947	-1.9684567	0	0.00450192
HALLMARK_ESTROGEN_RESPONSE_EARLY	74	-0.3420985	-1.8633506	0.006297229	0.01128497
HALLMARK_NOTCH_SIGNALING	12	-0.5283984	-1.6967478	0.017080745	0.03626061
HALLMARK_KRAS_SIGNALING_UP	55	-0.3314411	-1.678077	0.016795866	0.03843789
HALLMARK_GLYCOLYSIS	73	-0.3045951	-1.6702735	0.012269938	0.03787533
HALLMARK_MTORC1_SIGNALING	105	-0.2846448	-1.6422013	0.014319809	0.04296818
HALLMARK_INFLAMMATORY_RESPONSE	49	-0.3113645	-1.5160452	0.062337663	0.09539565
HALLMARK_ANDROGEN_RESPONSE	44	-0.2996694	-1.430291	0.08377659	0.14754075
HALLMARK_ALLOGRAFT_REJECTION	41	-0.300817	-1.4232167	0.09139785	0.14598949
HALLMARK_ESTROGEN_RESPONSE_LATE	57	-0.2622454	-1.3263744	0.14012739	0.22631517
HALLMARK_DNA_REPAIR	63	-0.2516645	-1.3182757	0.11720698	0.22502989
HALLMARK_PROTEIN_SECRETION	26	-0.3165565	-1.3127619	0.15714286	0.22081989
HALLMARK_P53_PATHWAY	81	-0.2302435	-1.2735578	0.15917376	0.25294754
HALLMARK_TGF_BETA_SIGNALING	30	-0.2885249	-1.2395627	0.2200282	0.28201476
HALLMARK_WNT_BETA_CATENIN_SIGNALING	24	-0.3065941	-1.2357075	0.21480407	0.27587956
HALLMARK_APOPTOSIS	59	-0.2299549	-1.1929116	0.24177215	0.31585085
HALLMARK_APICAL_SURFACE	12	-0.3485527	-1.1093022	0.35191083	0.41205016
HALLMARK_HYPOXIA	80	-0.2040796	-1.1059246	0.33616504	0.4030483
HALLMARK_UV_RESPONSE_DN	71	-0.1826215	-0.9771295	0.5124378	0.57752246
HALLMARK_REACTIVE_OXYGEN_SPECIES_PATHWAY	18	-0.2694655	-0.9603014	0.51780415	0.58443105
HALLMARK_ADIPOGENESIS	67	-0.1577214	-0.850439	0.67206985	0.732647
HALLMARK_COAGULATION	45	-0.1623898	-0.7809164	0.7642384	0.80532336
HALLMARK_OXIDATIVE_PHOSPHORYLATION	61	-0.1251184	-0.6650697	0.87913483	0.91074115
HALLMARK_SPERMATOGENESIS	27	-0.1523897	-0.6289954	0.9033189	0.91499346

ES, Enrichment score
NES, Normalised enrichment score
NOM p-value, Nominal p value
FDR q-value, False discovery rate
*Number of genes in the the gene set after filtering genes not in the expression dataset

Table S4 Preranked geneset enrichment analysis comparing PKC inhibitor- versus control-treated MP38 uveal melanoma cell line

NAME	SIZE*	ES	NES	NOM p-val	FDR q-val
HALLMARK_UV_RESPONSE_DN	43	0.39729747	2.496082	0	0
HALLMARK_MITOTIC_SPINDLE	46	0.29698822	1.8645478	0.002141328	0.07939816
HALLMARK_APICAL_JUNCTION	30	0.3285541	1.8265758	0.006681514	0.06551886
HALLMARK_INTERFERON_ALPHA_RESPONSE	12	0.40024826	1.4750972	0.077568136	0.3914901
HALLMARK_PI3K_AKT_MTOR_SIGNALING	31	0.2605358	1.4714326	0.07261411	0.3183224
YAP1_UP	12	0.3896555	1.4670557	0.08181818	0.2696664
HALLMARK_APOPTOSIS	46	0.22502099	1.4216101	0.06880734	0.29223317
HALLMARK_BILE_ACID_METABOLISM	16	0.33676732	1.3887641	0.116630666	0.3016677
HALLMARK_HEME_METABOLISM	48	0.2137625	1.3736396	0.11293635	0.28645095
HALLMARK_P53_PATHWAY	57	0.20210463	1.3608133	0.12195122	0.27245384
HALLMARK_TGF_BETA_SIGNALING	20	0.28080806	1.329926	0.13978495	0.2791529
HALLMARK_COAGULATION	21	0.28328523	1.3280321	0.15367965	0.25843593
HALLMARK_CHOLESTEROL_HOMEOSTASIS	22	0.26031688	1.2503935	0.19027483	0.32532638
HALLMARK_PEROXISOME	19	0.26910734	1.2207451	0.20474137	0.3407606
HALLMARK_MYOGENESIS	36	0.21336329	1.2178637	0.22736841	0.32181486
HALLMARK_ANDROGEN_RESPONSE	25	0.23555301	1.1983209	0.25373134	0.3252242
HALLMARK_EPITHELIAL_MESENCHYMAL_TRANSITION	47	0.17249943	1.1026704	0.325	0.43089578
HALLMARK_KRAS_SIGNALING_DN	16	0.23777708	1.0081325	0.43412527	0.5457183
HALLMARK_FATTY_ACID_METABOLISM	30	0.17195827	0.9291993	0.52796423	0.63468987
HALLMARK_APICAL_SURFACE	11	0.24986759	0.92192835	0.54968286	0.6144306
HALLMARK_XENOBIOTIC_METABOLISM	31	0.13304333	0.73676854	0.77896994	0.8403224
HALLMARK_PROTEIN_SECRETION	17	0.16092534	0.68690026	0.83027524	0.85850376
MAPK Signaling V2	39	-0.7913364	-4.610326	0	0
HALLMARK_MYC_TARGETS_V2	44	-0.661743	-3.9949946	0	0
MAPK Signaling V1	20	-0.7936011	-3.5738542	0	0
HALLMARK_MYC_TARGETS_V1	77	-0.4768841	-3.4484365	0	0
HALLMARK_TNFA_SIGNALING_VIA_NFKB	64	-0.4631783	-3.2017386	0	0
HALLMARK_E2F_TARGETS	51	-0.4772617	-2.9667883	0	0
HALLMARK_IL2_STAT5_SIGNALING	53	-0.3656089	-2.3517218	0	6.58E-04
HALLMARK_ESTROGEN_RESPONSE_EARLY	49	-0.3496517	-2.1835122	0	0.0022812
HALLMARK_COMPLEMENT	35	-0.3583342	-1.9872638	0.003846154	0.00812607
HALLMARK_G2M_CHECKPOINT	56	-0.3039371	-1.9196625	0.005681818	0.01300306
HALLMARK_KRAS_SIGNALING_UP	34	-0.3329577	-1.8667588	0.009398496	0.01735629
HALLMARK_UNFOLDED_PROTEIN_RESPONSE	35	-0.3385014	-1.8573638	0.003669725	0.01693923
HALLMARK_MTORC1_SIGNALING	76	-0.226357	-1.6358293	0.022514071	0.0582812
HALLMARK_HYPOXIA	62	-0.2363153	-1.60203	0.026548672	0.06596709
HALLMARK_OXIDATIVE_PHOSPHORYLATION	43	-0.2578217	-1.5316588	0.056179777	0.09117929
HALLMARK_DNA_REPAIR	32	-0.2867427	-1.5315754	0.056420233	0.0856058
HALLMARK_UV_RESPONSE_UP	41	-0.2589209	-1.5108522	0.06679035	0.08906081
HALLMARK_ESTROGEN_RESPONSE_LATE	45	-0.2042837	-1.2719527	0.18402778	0.2414117
HALLMARK_INFLAMMATORY_RESPONSE	31	-0.2255364	-1.2114923	0.24175824	0.28732982
HALLMARK_ALLOGRAFT_REJECTION	22	-0.2577669	-1.1811918	0.25765765	0.3046725
HALLMARK_GLYCOLYSIS	61	-0.1727058	-1.1625557	0.26915887	0.3109568
HALLMARK_REACTIVE_OXYGEN_SPECIES_PATHWAY	11	-0.3044539	-1.0428131	0.39259258	0.43744707
HALLMARK_ADIPOGENESIS	50	-0.1438014	-0.8959451	0.6	0.61705446
HALLMARK_INTERFERON_GAMMA_RESPONSE	26	-0.1803912	-0.8810523	0.6316726	0.61187893

ES, Enrichment score
NES, Normalised enrichment score
NOM p-value, Nominal p value
FDR q-value, False discovery rate
*Number of genes in the the gene set after filtering genes not in the expression dataset

Table S5 Preranked geneset enrichment analysis comparing PKC inhibitor- and control-treated MP46 uveal melanoma cell line

NAME	SIZE*	ES	NES	NOM p-val	FDR q-val
HALLMARK_CHOLESTEROL_HOMEOSTASIS	31	0.4290897	2.306397	0	0.00378968
HALLMARK_HEME_METABOLISM	63	0.2910889	2.024233	0	0.0285285
HALLMARK_HYPOXIA	77	0.263758	1.8930992	0.003067485	0.04445431
HALLMARK_BILE_ACID_METABOLISM	23	0.38900742	1.8605285	0.010282776	0.04138155
HALLMARK_PROTEIN_SECRETION	17	0.39220074	1.675445	0.03125	0.09808318
HALLMARK_UV_RESPONSE_DN	57	0.21457396	1.3858175	0.075075075	0.34726754
HALLMARK_PEROXISOME	26	0.27230853	1.3528056	0.12398922	0.3439819
HALLMARK_ESTROGEN_RESPONSE_LATE	48	0.19888447	1.2780358	0.1516035	0.4199991
HALLMARK_FATTY_ACID_METABOLISM	38	0.21105202	1.2242346	0.20775624	0.46064848
HALLMARK_MYOGENESIS	34	0.21957818	1.2109303	0.23450135	0.43586272
HALLMARK_OXIDATIVE_PHOSPHORYLATION	30	0.17391582	0.94078517	0.5257453	0.97710735
HALLMARK_MITOTIC_SPINDLE	36	0.16312295	0.92963624	0.5472222	0.9220528
YAP1_UP	12	0.24012789	0.883652	0.58468676	0.94859964
HALLMARK_XENOBIOTIC_METABOLISM	35	0.15175939	0.8753173	0.6440678	0.89824003
HALLMARK_KRAS_SIGNALING_DN	22	0.17630543	0.8267903	0.6819338	0.92828196
HALLMARK_APICAL_JUNCTION	42	0.1345912	0.8099711	0.73504275	0.89840084
HALLMARK_INTERFERON_ALPHA_RESPONSE	20	0.17890452	0.80955493	0.6984536	0.84599215
HALLMARK_REACTIVE_OXYGEN_SPECIES_PATHWAY	11	0.22364864	0.79789305	0.74004686	0.8170243
HALLMARK_SPERMATOGENESIS	14	0.19400105	0.7579023	0.7866005	0.82765085
HALLMARK_ADIPOGENESIS	51	0.11129022	0.7082461	0.8746594	0.8431531
MAPK Signaling V2	44	-0.8099839	-4.301441	0	0
MAPK Signaling V1	22	-0.8209446	-3.5960581	0	0
HALLMARK_MYC_TARGETS_V2	42	-0.5987147	-3.1308978	0	0
HALLMARK_TNFA_SIGNALING_VIA_NFKB	67	-0.4923871	-2.9484026	0	0
HALLMARK_MYC_TARGETS_V1	48	-0.5019445	-2.7679687	0	0
HALLMARK_IL2_STAT5_SIGNALING	62	-0.4172058	-2.4606175	0	1.44E-04
HALLMARK_COMPLEMENT	38	-0.4438678	-2.2434983	0	0.00128612
HALLMARK_E2F_TARGETS	34	-0.4433091	-2.180279	0	0.00236925
HALLMARK_UNFOLDED_PROTEIN_RESPONSE	34	-0.4232392	-2.0777402	0.001579779	0.00574645
HALLMARK_KRAS_SIGNALING_UP	42	-0.3664457	-1.9082967	0.001538462	0.01732734
HALLMARK_ESTROGEN_RESPONSE_EARLY	68	-0.3127505	-1.8663133	0.003021148	0.02171966
HALLMARK_INFLAMMATORY_RESPONSE	37	-0.3520363	-1.7678642	0.017883755	0.03652017
HALLMARK_APICAL_SURFACE	13	-0.4572346	-1.6191561	0.03529412	0.08189625
HALLMARK_G2M_CHECKPOINT	43	-0.3083535	-1.6066875	0.021138212	0.08208717
HALLMARK_UV_RESPONSE_UP	33	-0.3303617	-1.5914253	0.040584415	0.08320379
HALLMARK_COAGULATION	31	-0.3117209	-1.4907014	0.0608	0.13600357
HALLMARK_INTERFERON_GAMMA_RESPONSE	34	-0.292433	-1.4131132	0.097484276	0.18692973
HALLMARK_ALLOGRAFT_REJECTION	22	-0.3323771	-1.4076918	0.117741935	0.1799704
HALLMARK_EPITHELIAL_MESENCHYMAL_TRANSITION	55	-0.2409342	-1.3661352	0.12180451	0.20427172
HALLMARK_GLYCOLYSIS	56	-0.2255524	-1.2684414	0.20092024	0.29048106
HALLMARK_ANDROGEN_RESPONSE	39	-0.2430194	-1.2558959	0.19236884	0.28920186
HALLMARK_P53_PATHWAY	61	-0.2146185	-1.2492586	0.20765832	0.2835307
HALLMARK_ANGIOGENESIS	12	-0.3672811	-1.2491399	0.21964286	0.27125514
HALLMARK_APOPTOSIS	45	-0.1977161	-1.0777413	0.37386018	0.47284976
HALLMARK_TGF_BETA_SIGNALING	18	-0.2706057	-1.0672047	0.36290324	0.46889928
HALLMARK_HEDGEHOG_SIGNALING	10	-0.320671	-1.0525436	0.37900355	0.46995527
HALLMARK_MTORC1_SIGNALING	66	-0.1716411	-1.0309488	0.4136947	0.48213208
HALLMARK_WNT_BETA_CATENIN_SIGNALING	20	-0.2313992	-0.9567328	0.5221675	0.5675413
HALLMARK_PI3K_AKT_MTOR_SIGNALING	30	-0.2012552	-0.9552603	0.5080645	0.55037636
HALLMARK_IL6_JAK_STAT3_SIGNALING	13	-0.2112334	-0.7569824	0.76430976	0.79714197
HALLMARK_DNA_REPAIR	14	-0.1951632	-0.7313904	0.78194207	0.799836

ES, Enrichment score
NES, Normalised enrichment score
NOM p-value, Nominal p value
FDR q-value, False discovery rate
*Number of genes in the the gene set after filtering genes not in the expression dataset
