# Supplementary material for: Protein kinase inhibitor responses in uveal melanoma reflects a diminished dependency on PKC-MAPK signaling
Source: Cancer Gene Ther. 2022 Mar 29;29(10):1384–93. doi: 10.1038/s41417-022-00457-2 (PMC9576594; doi:10.1038/s41417-022-00457-2)
Supplement: Supplementary file 2 — Supplementary Figures [file 41417_2022_457_MOESM2_ESM.docx]

**
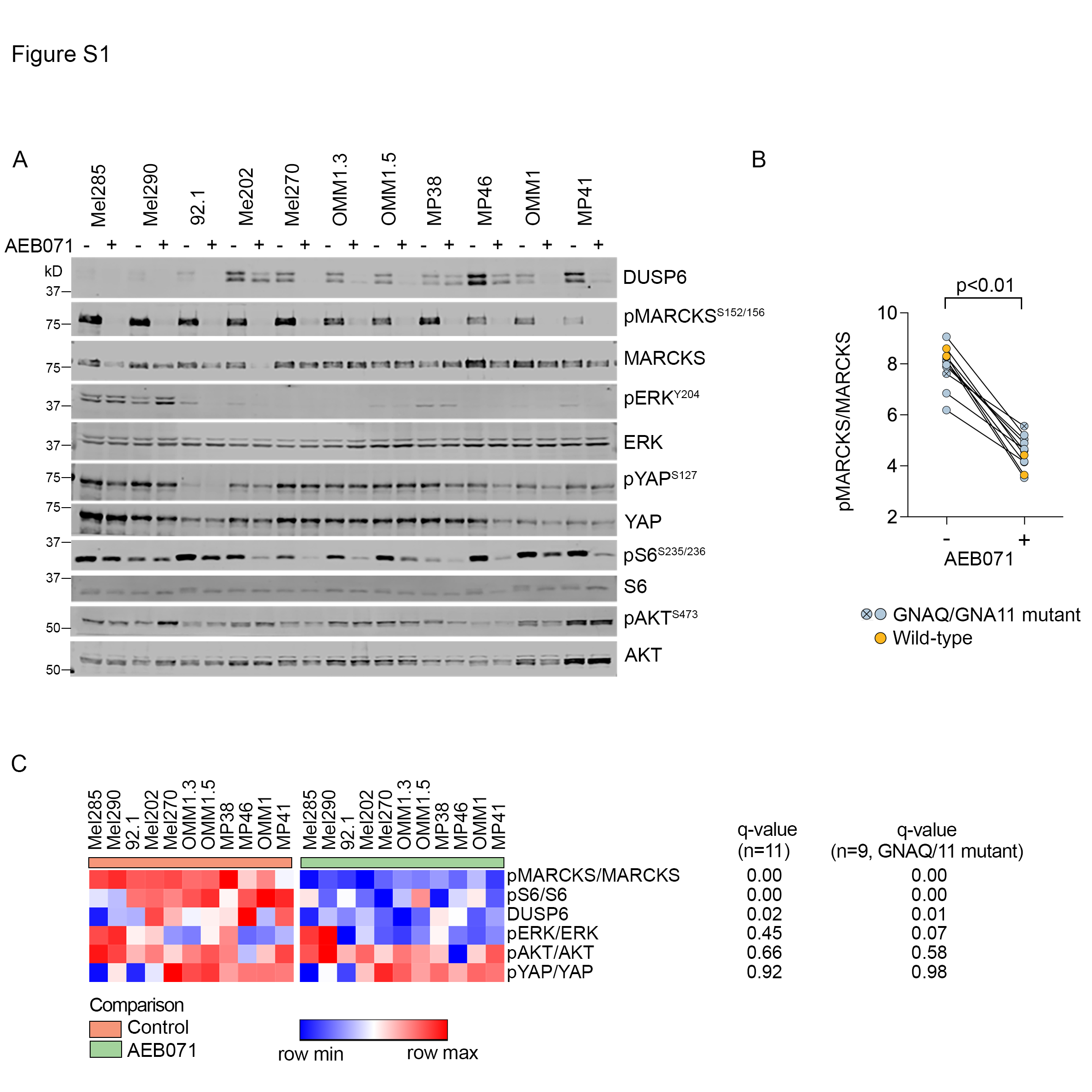
Supplementary Figure 1.** **AEB071 suppresses MARCKS phosphorylation in UM cell lines**

1. Protein expression of MAPK, PI3K, YAP and PKC signaling effectors after 24 h of treatment with BSA control (-) or 5µM AEB071 (+).
2. Normalised levels of pMARCKS were compared pre (-) and post (+) PKC inhibitor treatment using the paired t-test. Normalised MARCKS protein expression is derived from an average of 2-3 independent experiments for each cell line. The GNAQ/GNA11 mutant Mel270 and OMM1 cells are highlighted by the crossed, circle symbol. The WT Mel285 and Mel290 UM cells are highlighted in orange. ns, not significant
3. Heatmap showing comparison of the indicated normalised proteins in control-treated vs AEB071-treated (24 h) UM cell lines. The false discovery (FDR) adjusted p-values (q-value) are shown for the comparison of control and treated UM cell lines, n=11 and *GNAQ/GNA11* mutant UM cell lines, n=9, using the t-test within the Morpheus web-based tool (<https://software.broadinstitute.org/morpheus/>)

**
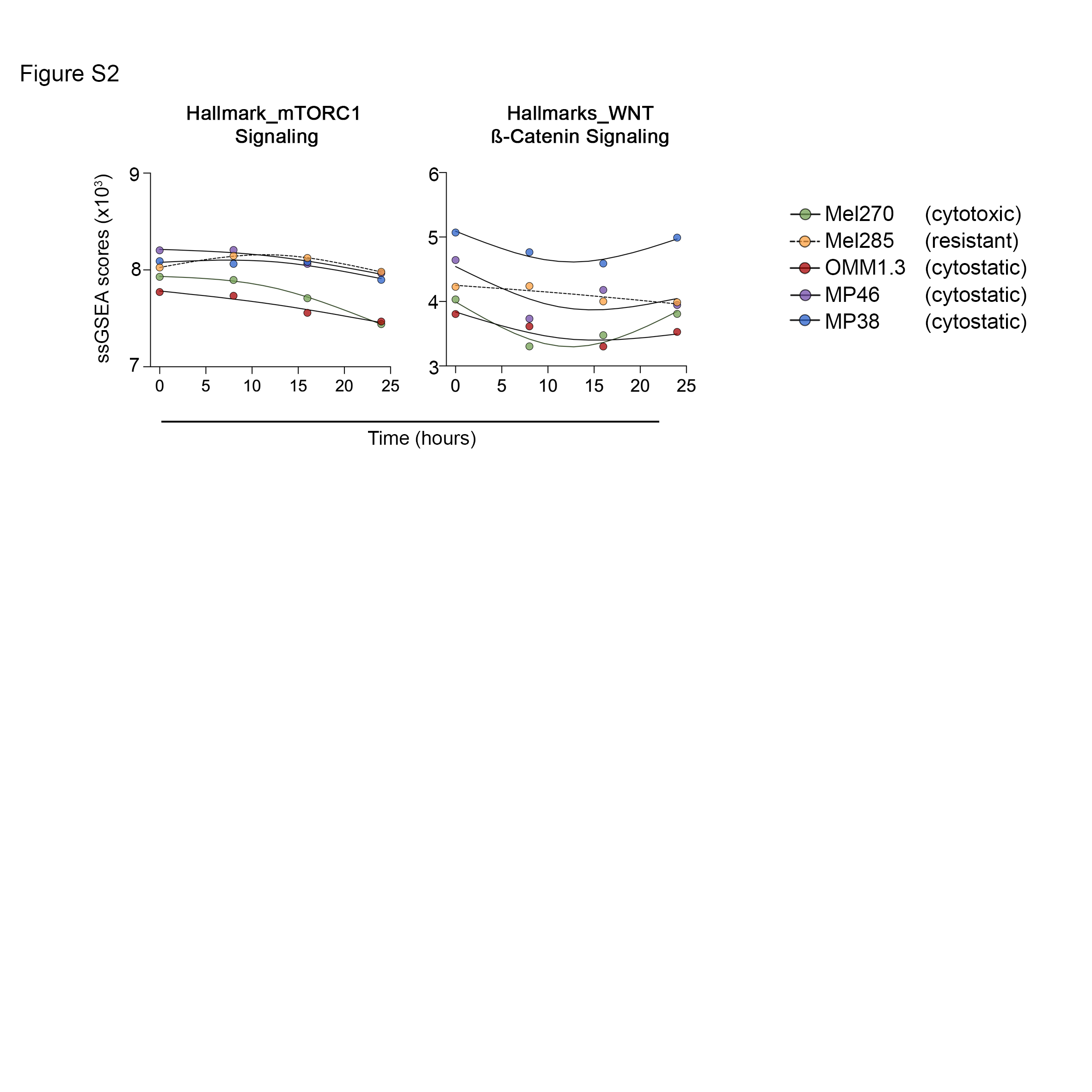
Supplementary Figure 2. mTORC and WNT-ß-catenin genesets do not show durable inhibition in response to PKC inhibition in UM cells**

Single sample gene set enrichment analysis (ssGSEA) scores for the indicated genesets are plotted over IDE196 inhibitor treatment time

**
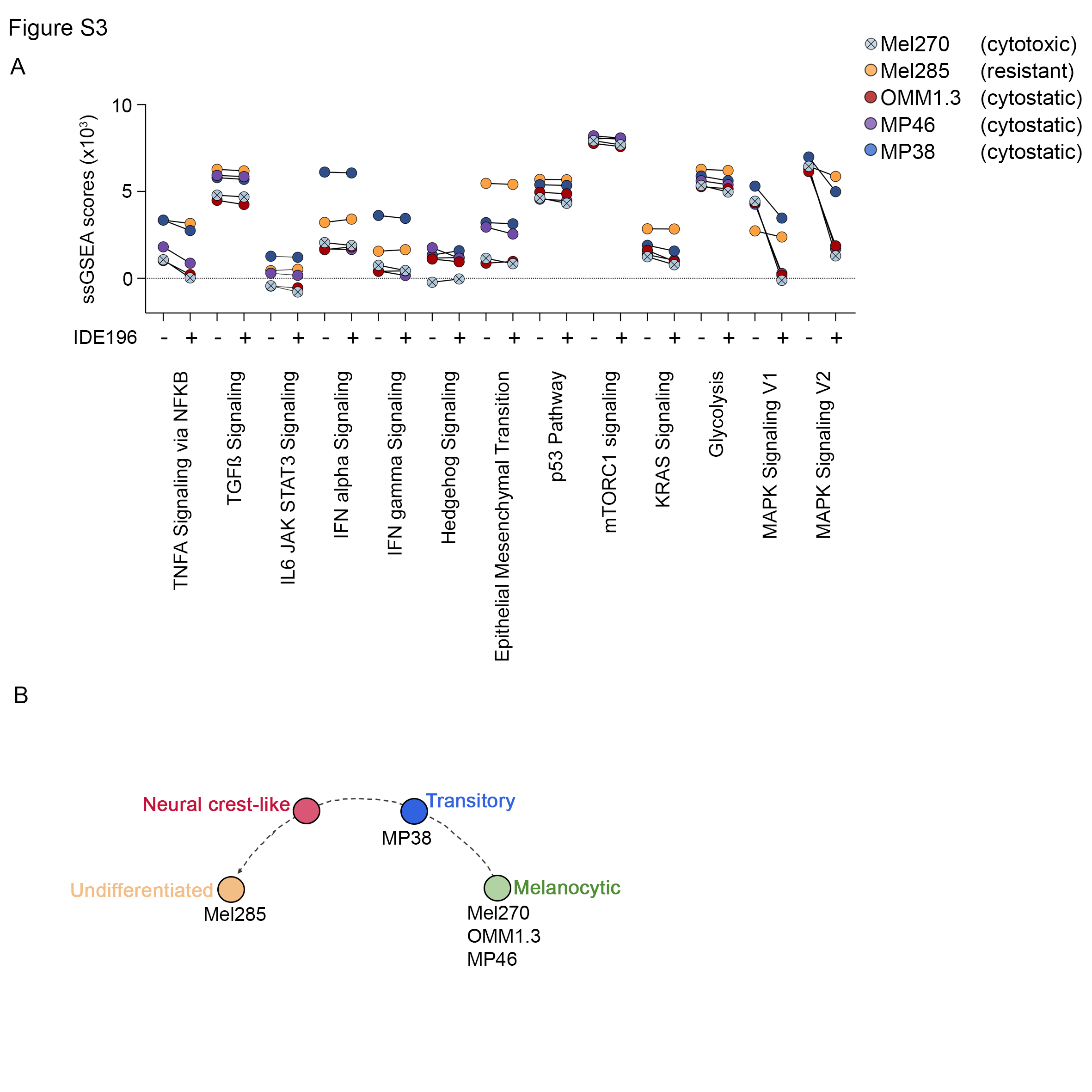
Supplementary Figure 3. Differentiation status of UM cell lines status pre and post PKC inhibition with IDE196**

1. Single sample gene set enrichment analysis (ssGSEA) scores for the indicated genesets for control (-) and IDE196 (+) treated UM cell lines. Each point represents average ssGSEA scores for 8 h, 16 h and 24 h time points
2. Schematic showing the UM transcriptome-based differentiation subtypes, along the four-step progressive differentiation trajectory
